# Supplementary material for: Progression of Thoracic Aortic Dissection Is Aggravated by the hsa_circ_0007386/miR-1271-5P/IGF1R/AKT Axis via Induction of Arterial Smooth Muscle Cell Apoptosis
Source: Biomedicines. 2023 Feb 15;11(2):571. doi: 10.3390/biomedicines11020571 (PMC9953311; doi:10.3390/biomedicines11020571)
Supplement: Supplementary file 1 [file biomedicines-11-00571-s001.zip › biomedicines-2141676-supplementary.pdf]

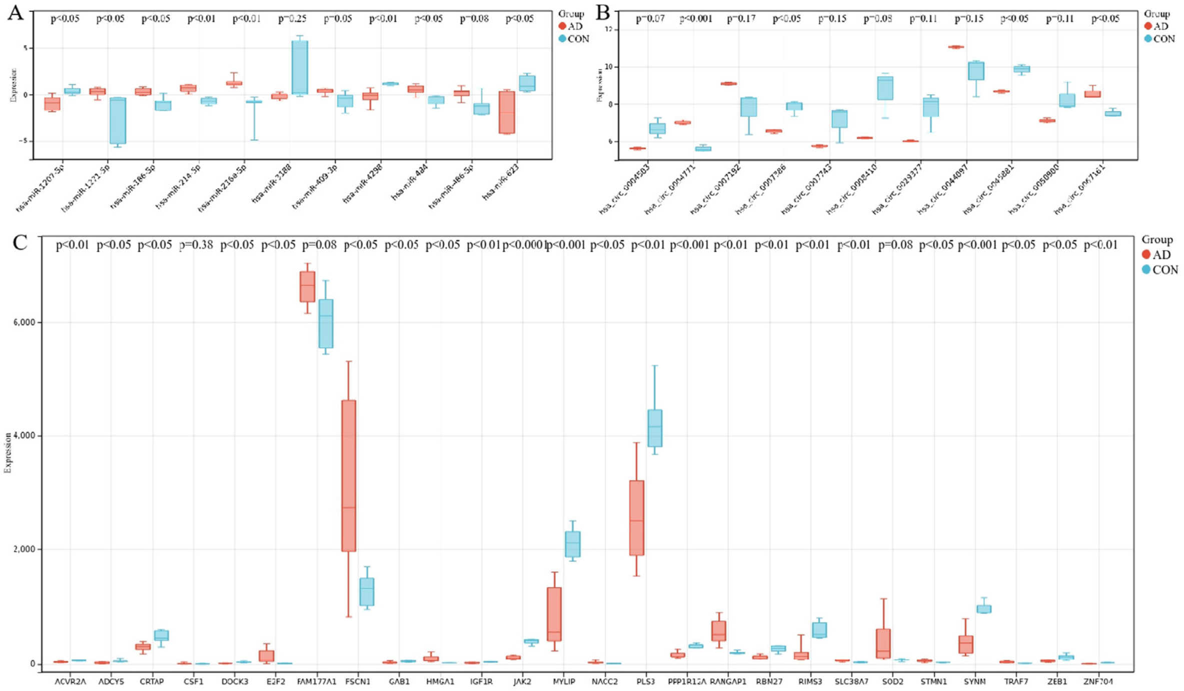

Supplementary Figure S1. The expression levels of the ce-cicRNA (A), ce-miRNA (B) and ce-mRNA (C) between the TAD and CON group in the GSE97741, GSE98770, and GSE52093 datasets respectively.

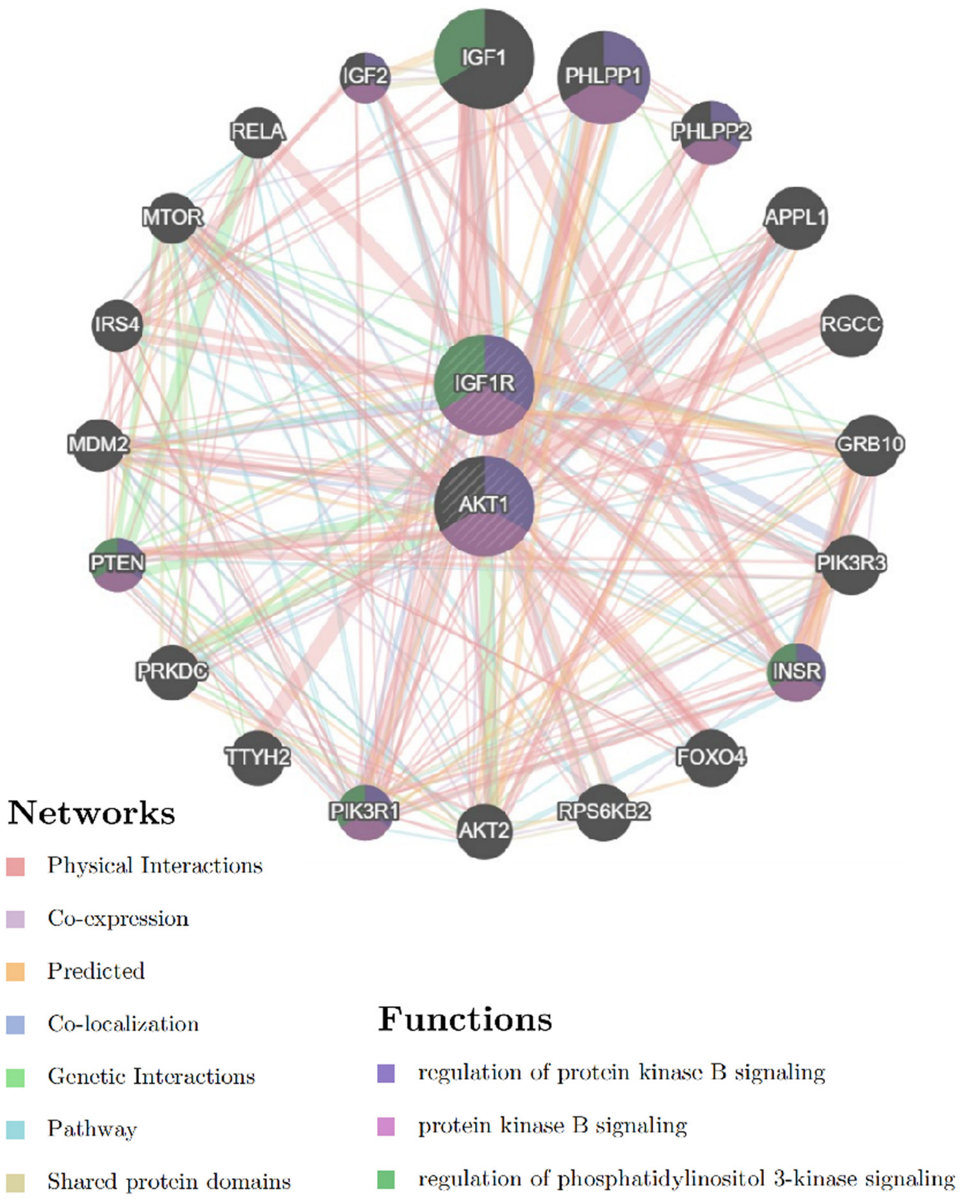

Supplementary Figure S2. GENEMANIA database shows the interaction between IGF1R and AKT.

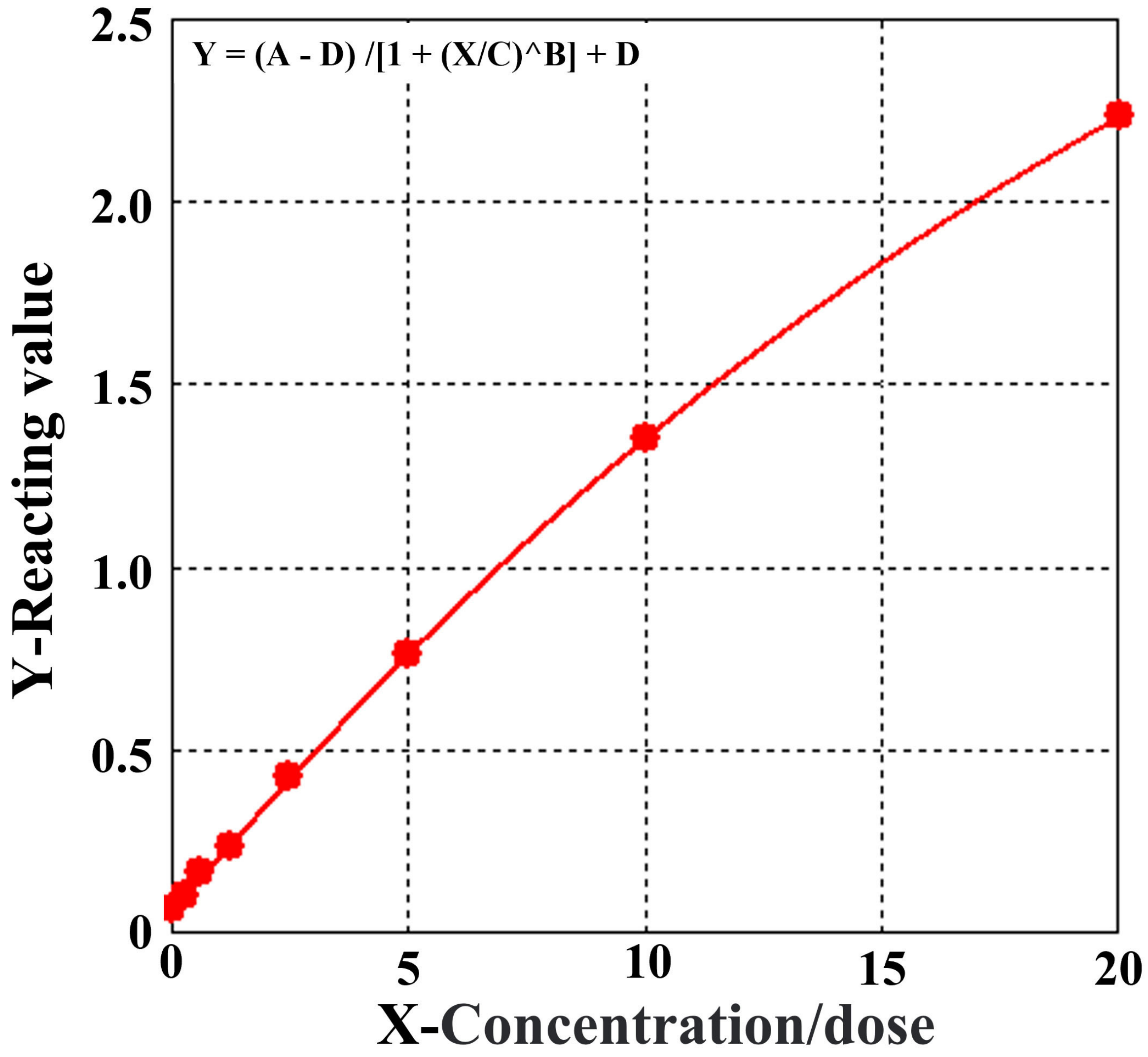

Supplementary Figure S3. Fitting Logistic curve of ELISA experiment.

Supplementary method

Immunohistochemistry (IHC)

Paraffin sections containing sufficient formalin fixed arterial wall tissue were sectioned continuously at a thickness of 4 μm and were mounted on silage coated slides for immunohistochemical analysis. The slices were deparaffinized with xylene and rehydrated in 95%, 85% and 75% ethanol. Antigen retrieval was performed by subjecting the slides to high-pressure sterilization at 121° C for 2 min in 0.01 mol/L sodium citrate buffer solution (pH 6.0). Endogenous peroxidase activity was blocked by incubating the slides with 3% H_2O_2 at room temperature for 10 min. The slices were then washed in phosphate buffered saline

(PBS) solution and blocked in 10% goat serum (Zhongshan Biotechnology Co, Ltd.) for 30 minutes. Next, the sections were incubated with diluted rabbit anti-human IGF1R (ab263903 diluted 1: 500.USA) overnight in a humidified chamber at 4° C. After three washes in PBS, the sections were incubated with the secondary antibody conjugated to horseradish peroxidase at room temperature for 30 minutes. The signal was developed with diaminobenzidine solution, which was followed by counterstaining in 20% hematoxylin. Finally, all slides were dehydrated and mounted on cover glass. For negative controls, antibody diluent was substituted for the primary antibody.

Western blot

Total protein was extracted from arterial wall tissue with RIPA lysis buffer (Biyotime, China) containing protease inhibitors. The protein concentration of the lysates was analyzed by BCA protein assays (Thermo Fisher Scientific, USA). 40 μg of protein was separated on a 10% SDS-polyacrylamide gel and blotted onto polyvinylidene difluoride (PVDF) membranes (Millipore, USA). After blocking with 5% bovine serum albumin (BSA) for 1 h, the membranes were then incubated with primary antibodies [IGF1R (ab263903, Abcam, USA); AKT (ab8805, abcam, USA); CASPASE3 (ab4051, Abcam, USA)] overnight at 4° C and horseradish peroxidase-conjugated secondary antibodies for 1 h at room temperature. Immunoreactive signals were detected using the ECL detection system. Immunoblotting of glyceraldehyde-3-phosphate dehydrogenase (GAPDH) was performed as an internal control.
